# Prophylactic role of omega 3 fatty acids in bisphenol F-induced sexual and erectile dysfunction is associated with penile redox homeostasis

**DOI:** 10.1016/j.bbrep.2025.102103

**Published:** 2025-06-23

**Authors:** Adeyemi Fatai Odetayo, Moses Agbomhere Hamed, Grace Edet Bassey, Oluranti Olayinka Titiloye, Samson Daniel Maduabuchi, Kazeem Bidemi Okesina, Luqman Aribidesi Olayaki

**Affiliations:** a Department of Physiology, Faculty of Basic Medical Sciences, Federal University of Health Sciences, Ila-Orangun, Nigeria; b Endocrinology, Reproductive, and Metabolism Unit, Physiology Department, Federal University of Health Sciences, Ila-Orangun, Nigeria; c Department of Medical Laboratory Science, Afe Babalola University, Ado-Ekiti, Nigeria; d The Brainwill Laboratories and Biomedical Services, Osogbo, Nigeria; e Physiology Department, Faculty of Basic Medical Sciences, University of Uyo, Uyo, Nigeria; f Department of Anatomy and Cell Biology, Obafemi Awolowo University, Ife, Nigeria; g Department of Medical Laboratory Sciences, Leeds City University, Ibadan, Nigeria; h Department of Physiology, University of Rwanda, Kigali, Rwanda; i Department of Physiology, University of Ilorin, Ilorin, Nigeria

**Keywords:** Bisphenol F, Endocrine disruptor, Nitric oxide (NO)/Cyclic guanosine monophosphate (cGMP), Penile erection, Penile redox imbalance, Xanthine oxidase/uric acid signaling

## Abstract

**Introduction:**

Bisphenol F (BPF) is an established environmental pollutant that has been shown to distort erectile function. However, the effect of BPF on penile redox homeostasis is not known. On the other hand, omega-3 fatty acids (O3FA) are known antioxidants with fertility-enhancing properties. Despite these abilities, the ameliorative effect of O3FA on BPF-induced penile redox imbalance is unknown. Hence, this study was designed to add to the existing body of knowledge by investigating the role of penile redox homeostasis on BPF-induced erectile dysfunction and the possible ameliorative effect of O3FA.

**Methodology:**

Twenty male Wistar rats were randomly divided into four groups (n = 5/group) after two weeks acclimatization period as follows: control group (.5 ml of corn oil), O3FA group (300mg/kg of O3FA), BPF group (30mg/kg of BPF), and the BPF + O3FA group (30 mg/kg of BPF + 300mg/kg of O3FA).

**Results:**

BPF exposure led to sexual and erectile dysfunction, which was accompanied by a disruption in erectogenic enzymatic activities and circulatory testosterone. These events were accompanied by the down-regulation of penile NO/cGMP signaling, oxido-inflammatory response, and penile histoarchitecture distortion. These observed BPF-induced distortions were ameliorated in animals that received O3FA co-treatment with BPF.

**Conclusion:**

BPF-induced erectile dysfunction was associated with penile redox imbalance, and O3FA ameliorated BPF-induced penile toxicity.

## Highlights

• BPF impaired male erectile functions.
• This toxic effect is associated with penile redox imbalance and accompanied by XO/UA signaling.
• O3FA attenuated BPF-induced sexual and erectile dysfunction.

## Graphical abstract

**Figure:**
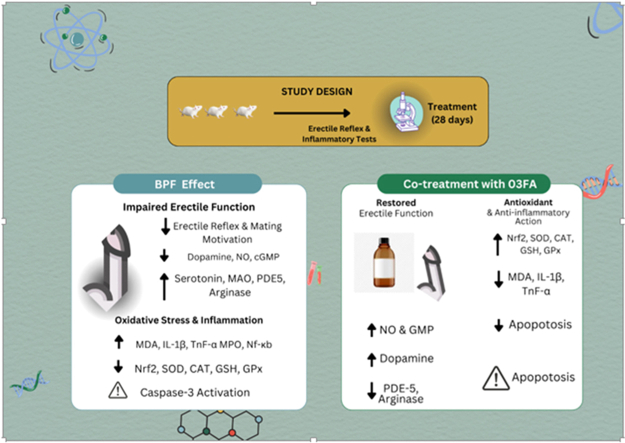

## 1 INTRODUCTION

Endocrine-disrupting chemicals (EDCs) are environmental toxicants that interfere with the functions of the endocrine system [1]. According to the definition provided by the Environmental Protection Agency (EPA), “EDCs are chemicals that disrupt the synthesis, secretion, transportation, binding or elimination of natural hormones in the body that are responsible for the maintenance of homeostasis, reproduction, development and/or behavior” [2]. Bisphenol F (BPF) is an environmental toxicant that falls under the category of EDCs [3]. It is largely utilized in the chemical, cosmetics, food, and pharmaceutical industries (Odetayo et al., [4,5]). BPF is increasingly recognized as a prevalent environmental contaminant linked to various stages of plastic production and disposal [6]. In addition to foodborne exposure, humans can also be exposed to BPF via inhalation and dermal contact, resulting in widespread detection in biological samples. Like other EDCs, BPF has been shown to interfere with the functions of the endocrine system, thus, disrupting sexual functions.

According to the report of our previous study [7], BPF impaired male sexual function by downregulating the nitric oxide (NO)/cyclic guanosine monophosphate (cGMP) signaling. However, the role of penile redox homeostasis in BPF-induced NO/cGMP signaling-mediated sexual dysfunction is yet to be discovered (Supplementary Table 1).

Oxidative stress is a major pathogenic condition that is possibly associated with penile dysfunction. Excessive reactive oxygen species (ROS) production has been shown to damage lipids, DNA, and protein in tissues [8]. Also, these ROS have been established to disrupt different cellular signal transduction such as the NO-mediated signaling [9]. Hence, it is conceivable that oxidative stress contributes to the disruption of penile vascular homeostasis, since conditions such as vasculogenic erectile dysfunction (ED), recurrent ischaemic priapism, and penile fibrotic that are associated with penile dysfunction are associated with NO-signaling [10]. For example, vasculogenic ED is a vascular endothelial disorder majorly associated with a disrupted endothelial NO-dependent mediatory pathway [11]. This disrupted NO-mediated signaling could result from the ability of the ROS to restrict the release and function of endothelial NO in the penis. Similarly, oxidative stress could prevent the release and actions of NO in recurrent ischaemic priapism, thereby leading to a prolonged erectile response [12]. Hence, it is plausible to hypothesize that ROS plays a key role in restricting the release and/or actions of NO in the penile tissue, thereby disrupting the NO/cGMP signaling.

On the contrary, omega-3 fatty acids (O3FAs) are essential polyunsaturated fatty acids that are majorly obtained from diet. There are three main types of O3FAs which include docosahexaenoic acid (DHA), alpha-linolenic acid (ALA), and eicosapentaenoic acid (EPA). EPA and DHA which are the major types of O3FAs are also known precursors of different metabolites regarded as potent lipid mediators, identified to be potent for preventing and treating different disorders [13]. For example, O3FAs have been shown to improve cardiovascular [14], testicular (Jensen et al., 2020 [15]), and erectile (Shim et al., 2016 [7]) functions. These properties of O3FAs are associated with their antioxidant and anti-inflammatory activities [16]. Hence, the cytoprotective effect of O3FAs against extrinsic toxic stimuli such as BPF could be associated with their antioxidant properties. Therefore, this study was designed to explore the antioxidant activities of O3FAs on BPF-induced penile redox imbalance.

## 2 METHODOLOGY

### 2.1 Ethical consideration

The experiment was designed in line with the National Institute of Health Guide for the Care and Use of Laboratory Animals guidelines, and the research protocol was approved by the Animal Care and Use Research Committee (ACURC), Faculty of Basic Medical Sciences, Federal University of Health Sciences, Ila-Orangun, Nigeria (FUHSI/BMS/AREC/2402). Furthermore, the Animal Research: Reporting of In Vivo Experiments (ARRIVE) guidelines for reporting experimental findings were followed strictly.

### 2.2 Experimental design

Twenty male Wistar rats of about 10 weeks old and similar weights were randomly divided into four groups (n = 5/group) after two weeks acclimatization period as follows; the control group that received .5 ml of corn oil, the O3FA group that received 300 mg/kg of O3FA, BPF group that were exposed to 30 mg/kg of BPF, and the BPF + O3FA group that were exposed to 30 mg/kg of BPF and co-treated with 300 mg/kg of O3FA. The dose of BPF used in this study is the same as what we've earlier reported [17,18] and it is similar to the dosage used and reported by Higashihara et al. [19] and Huitao et al. [20]. Also, that of O3FA is similar to what has been earlier reported from our laboratory ([21]; Odetayo et al., [4,5]).

### 2.3 Assessment of sexual and erectile function

The animals were kept in transparent cages (one male animal per cage) and a receptive female was introduced into each of the cages under a dim light and in a quiet room 12 h after the last dose of the drugs was administered. The female rats were made receptive artificially via the subcutaneous injection of progesterone (0.5 mg/100 g body weight) and estradiol benzoate (10 mg/100 g body weight) 4 h and 48 h respectively before pairing [22]. Receptivity was confirmed using physical assessment and vaginal smear [17] and only the receptive animals were paired with the male rats.

The non-contact penile erection was estimated according to established methods [23]. The male animals were kept in a clean cabin that was divided into half by sheets of plastic fiber mesh. The partition prevented contact but allowed auditory, visual, and olfactory stimuli. A mirror was strategically placed in order to record and view the erections in the ventral and lateral positions, and the penile visibility from the penile sheath was recorded as the number of penile erection and scored as penile erection X mean number of erections. The contact penile erection was estimated as previously established [24]. Each of the male rats was laid in a supine position with minimal restraint and the preputial sheath was retracted behind the glands for 15 min to illicit genital reflexes. The total penile reflexes (an index of penile erection and function) were determined as the sum of erection, long flips, and quick flips.

Motivation to mate, an index of libido was determined as previously established [7,17]. The male sexual behavior was monitored for 30 min using a camcorder and motivation to mate was scored as below “0: no sexual activity, 1: no interaction, rears and climbs on the chamber, 2: sniffs the female rat, 3: self-exploratory behavior such as grooming and sniffing of genitals, 4: grooms female counterpart anywhere 5: rears and climbs sexually, 6: pursues and sniffs the female rat, 7: tries to mount but easily discouraged, 8: mounts with an integrated deliberate manner and not easily discouraged, 9: reflex and almost involuntary mount”.

### 2.4 Sample collection

After the experimental duration, the animals were fasted overnight and euthanized with an intraperitoneal injection of 40mg/kg ketamine and 4mg/kg xylazine in the morning. The blood samples were collected by cardiac puncture into heparinized sample bottles and plasma was separated by centrifuging the blood sample at 3000 for 5 min. The penile tissue was also collected, separated from the surrounding structures, and weighed. The cavernosal tissue was removed, homogenized, and centrifuged at 10,000 for 15minat 40C to obtain supernatant.

### 2.5 Assessment of NO/cGMP signaling and erectogenic enzymes

Penile NO and cGMP were determined with the standard ELISA kits (Biovision Research Products, USA, and Elabscience Biotechnology Inc., USA respectively). Also, Dopamine (Abnova, UK) and serotonin (Elabscience, US) concentration and acetylcholinesterase (AchE) activities (Elabscience, US) were estimated using an ELISA kit (and manufacturer's guidelines were strictly followed. Monoamine oxidase (MAO) was estimated based on already established colorimetry method [25]. Additionally, phosphodiesterase-5, PDE5, and arginase activities were determined using the colorimetric method as previously established by Kelly and Butler [26] and Zhang et al. [27].

### 2.6 Oxidative stress, inflammatory, and apoptotic markers

Penile malondialdehyde (MDA) was determined using colorimetry methods and the manufacturer's (Oxford Biomedical Research, Inc., Oxford, USA) guidelines were strictly followed. Reduced glutathione (GSH) was also estimated based on the method of Beutler et al. [28]. Furthermore, penile superoxide dismutase (SOD) and catalase (CAT) activities were determined based on established methods [15,29,30] respectively. Penile levels of interleukin 1 beta (IL-1β), tumor necrosis alpha (TNF-α), and caspase 3 were determined using the ELISA method (Elabscience, USA) while penile MPO was estimated based on the method of Desser et al [31]. Also, penile Nrf2 Nrf2 and NF-κB were determined using an ELISA method (Elabscience, USA). In addition, penile xanthine oxidase (XO) and uric acid (UA) were determined using method methods, and the manufacturer's (Fortress Diagnostic, Antrim, UK and Precision Kit, India respectively) were strictly followed.

### 2.7 Histological processing

Also, the penile histology was performed as previously described. “Briefly, the penis was fixed in bouin solution and was dehydrated with ethanol series cleared with toluene, embedded at room temperature, and blocked in paraffin wax. The Hematoxylin and Eosin (H&E) stain was then applied to the 5 μm thick paraffin sections of the penis”.

## 3 Results

**Fig. 1:**
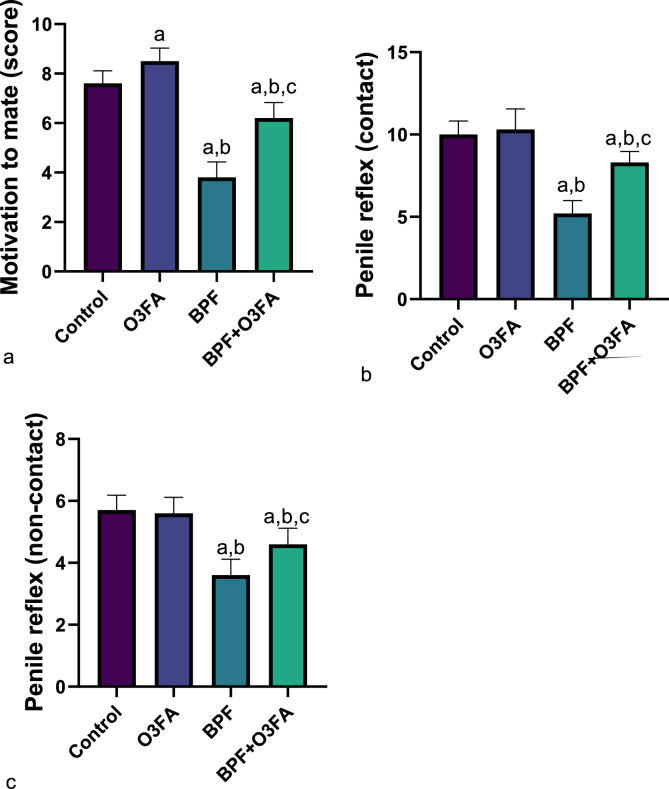
Effect of omega 3 fatty acids on (a) motivation to mate (b) penile reflex (contact) (c) penile reflex (non-contact) in BPF-exposed rats. ^a^p <0 .05 versus control, ^b^p < 0.05 versus O3FA, ^c^p < 0.05 versus BPF using one-way analysis of variance (ANOVA) followed by Tukey's post hoc test for pairwise comparison. BPF: Bisphenol F, O3FA: omega-3 fatty acids.

**Fig. 2:**
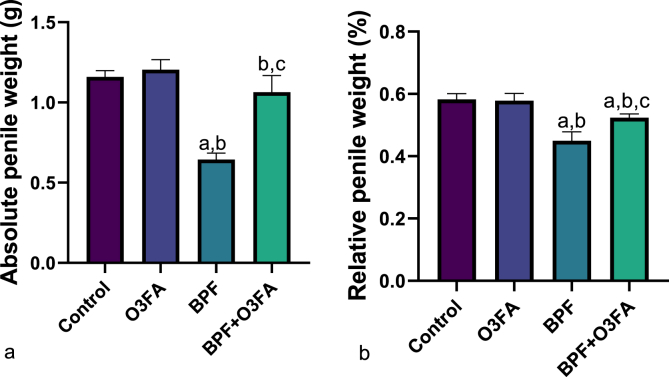
Effect of omega 3 fatty acids on (a) absolute penile weight (b) relative penile weight in BPF-exposed rats. ^a^p <0 .05 versus control, ^b^p < 0.05 versus O3FA, ^c^p < 0.05 versus BPF using one-way analysis of variance (ANOVA) followed by Tukey's post hoc test for pairwise comparison. BPF: Bisphenol F, O3FA: omega-3 fatty.

As shown in Fig. 1, BPF exposure significantly impaired male sexual functions evidenced by a significant decrease in motivation to mate and penile reflex (contact and non-contact) when compared with animals in the control group. These significant differences were ameliorated in the O3FA and BPF co-treated group. Additionally, BPF exposure significantly reduced the absolute and relative penile weight of animals in the BPF-exposed group compared to their counterparts in the negative and positive control groups (Fig. 2). These reductions were blunted in animals that received BPF and O3FA co-treatment.

**Fig. 3:**
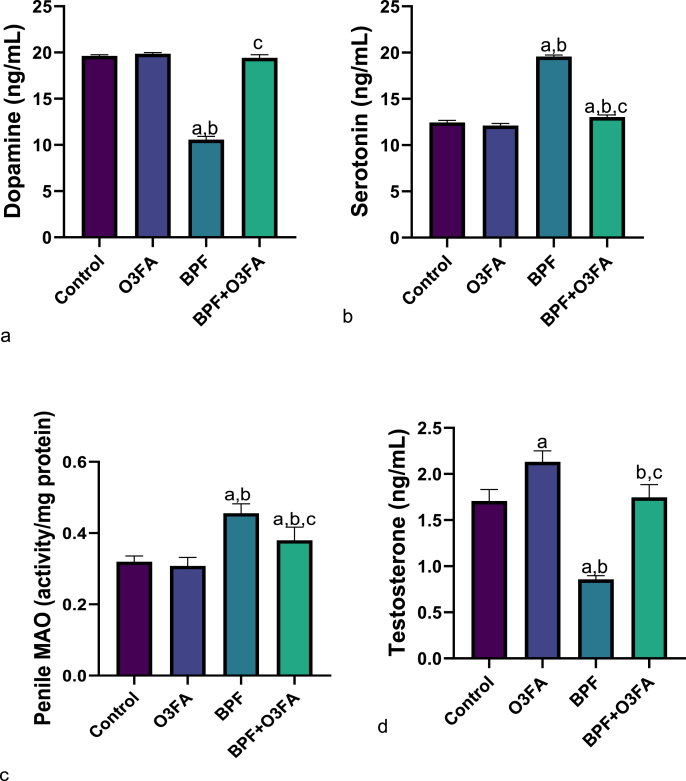
Effect of omega 3 fatty acids on (a) dopamine (b) serotonin (c) penile monoamine oxidase (MAO) (d) testosterone in BPF-exposed rats. ^a^p <0 .05 versus control, ^b^p < 0.05 versus O3FA, ^c^p < 0.05 versus BPF using one-way analysis of variance (ANOVA) followed by Tukey's post hoc test for pairwise comparison. BPF: Bisphenol F, O3FA: omega-3 fatty acids.

Furthermore, there was a significant decrease in dopamine but an increase in serotonin and MAO (Fig. 3) in BPF-exposed rats compared to their counterparts in both control groups. Also, BPF exposure led to a significant decrease in testosterone compared with the control animals. These observed differences were ameliorated in animals in the BPF + O3FA group.

**Fig. 4:**
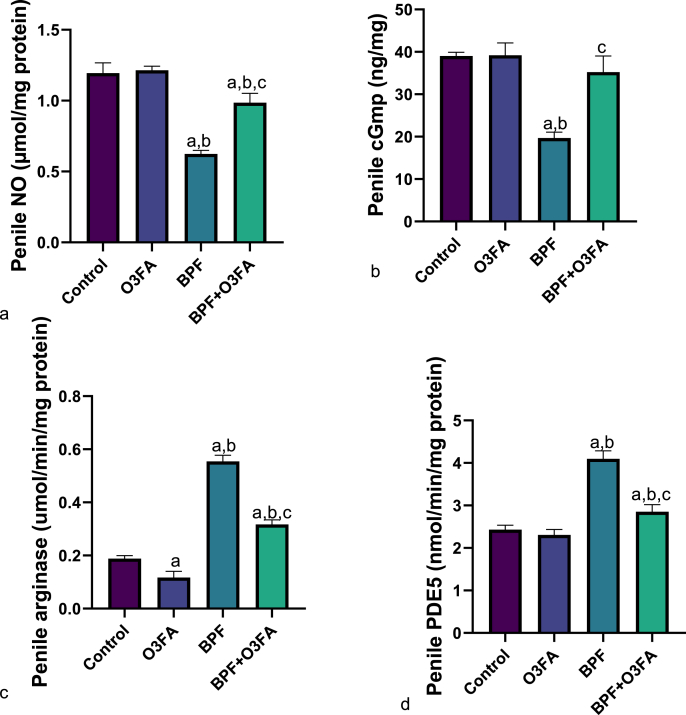
Effect of omega 3 fatty acids on (a) penile nitric oxide (NO) (b) penile cyclic guanosine monophosphate (cGMP) (c) penile monoamine oxidase (MAO) (d) penile phosphodiesterase type 5 (PDE5)in BPF-exposed rats. ^a^p <0 .05 versus control, ^b^p < 0.05 versus O3FA, ^c^p < 0.05 versus BPF using one-way analysis of variance (ANOVA) followed by Tukey's post hoc test for pairwise comparison. BPF: Bisphenol F, O3FA: omega-3 fatty acids.

Additionally, there was a significant decrease in penile NO and cGMP (Fig. 4) and an increase in arginase and PDE5 in animals exposed to BPF compared with their counterparts in the control groups. These observed alterations were abolished in animals that received BPF and O3FA co-treatment.

**Fig. 5:**
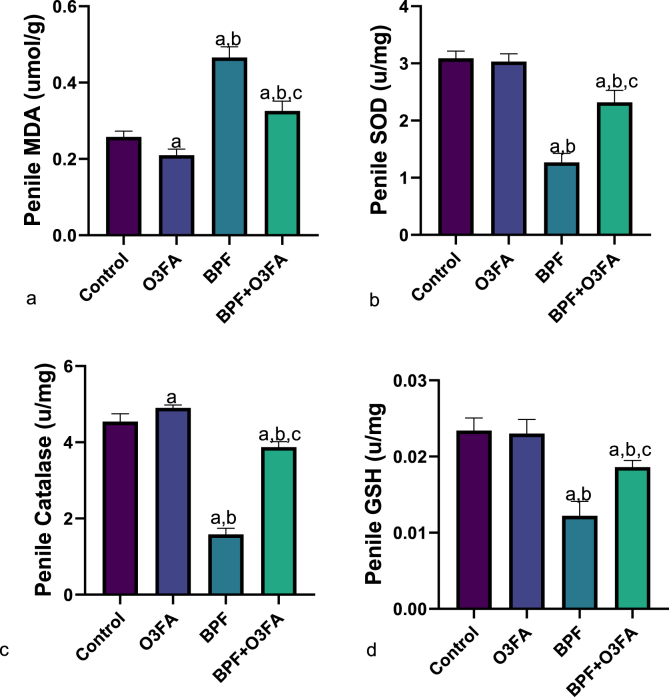
Effect of omega 3 fatty acids on (a) penile malondialdehyde (MDA) (b) penile superoxide dismutase (SOD) (c) penile catalase (d) penile glutathione (GSH) in BPF-exposed rats. ^a^p <0 .05 versus control, ^b^p < 0.05 versus O3FA, ^c^p < 0.05 versus BPF using one-way analysis of variance (ANOVA) followed by Tukey's post hoc test for pairwise comparison. BPF: Bisphenol F, O3FA: omega-3 fatty acids.

**Fig. 6:**
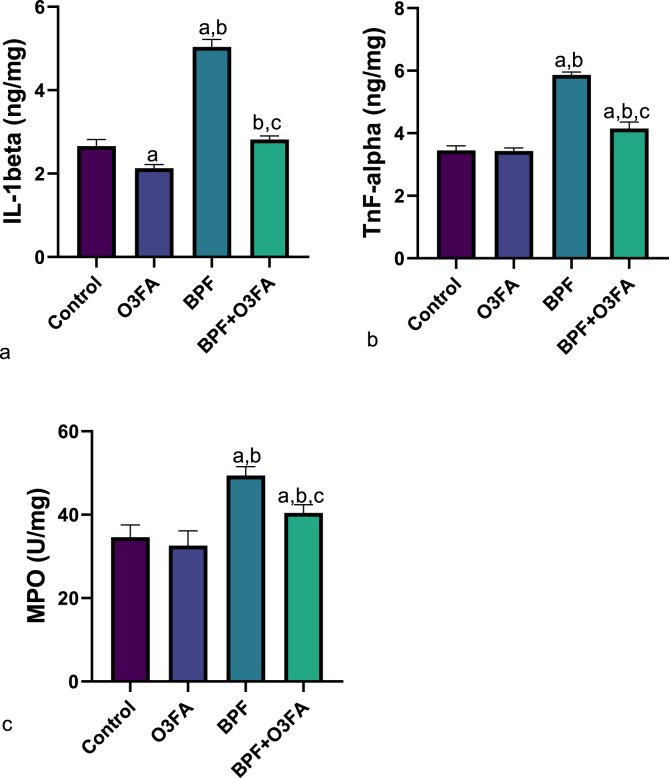
Effect of omega 3 fatty acids on penile (a) interleukin 1 beta (IL-1beta) (b) tumor necrosis factor alpha (Tnf-alpha) (c) Myeloperoxidase (MPO) in BPF-exposed rats. ^a^p <0 .05 versus control, ^b^p < 0.05 versus O3FA, ^c^p < 0.05 versus BPF using one-way analysis of variance (ANOVA) followed by Tukey's post hoc test for pairwise comparison. BPF: Bisphenol F, O3FA: omega-3 fatty acids.

**Fig. 7:**
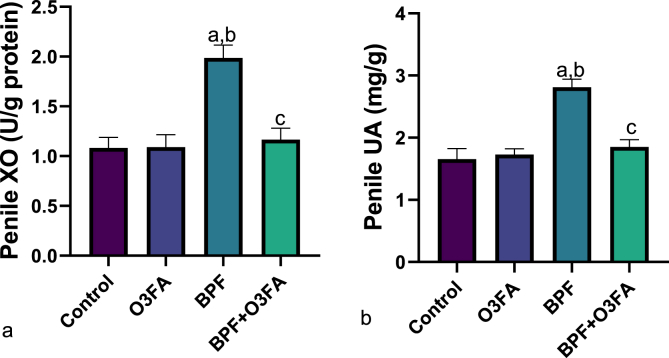
Effect of omega 3 fatty acids on penile (a) xanthine oxidase (XO) (b) uric acid (UA) in BPF-exposed rats. ^a^p <0 .05 versus control, ^b^p < 0.05 versus O3FA, ^c^p < 0.05 versus BPF using one-way analysis of variance (ANOVA) followed by Tukey's post hoc test for pairwise comparison. BPF: Bisphenol F, O3FA: omega-3 fatty acids.

**Fig. 8:**
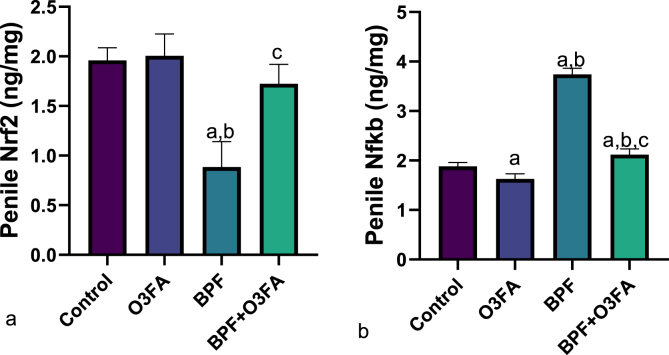
Effect of omega 3 fatty acids on penile (a) nuclear factor erythroid 2-related factor 2 (b) nuclear factor kappa B (Nfkb) in BPF-exposed rats. ^a^p <0 .05 versus control, ^b^p < 0.05 versus O3FA, ^c^p < 0.05 versus BPF using one-way analysis of variance (ANOVA) followed by Tukey's post hoc test for pairwise comparison. BPF: Bisphenol F, O3FA: omega-3 fatty acids.

Also, a significant increase in penile MDA and decrease in SOD, CAT, and GSH were observed in animals in the BPF group compared with the control groups (Fig. 5), while the observed increase in oxidative stress markers was abrogated in animals co-treated with BPF and O3FA. In the same vein, BPF exposure led to a significant increase in penile IL-1beta, TnF-α, and MPO compared with the control animals (Fig. 6). These observed increases in inflammatory markers were ameliorated in animals in the BPF + O3FA group. Also, the observed increase in oxidative stress and inflammatory markers in BPF-exposed animals was accompanied by a significant decrease in penile Nrf2 (Fig. 7) and an increase in penile XO, UA, and Nf-kb (Fig. 8). Similarly, these observed alterations were blunted in animals in the BPF + O3FA group.

**Fig. 9:**
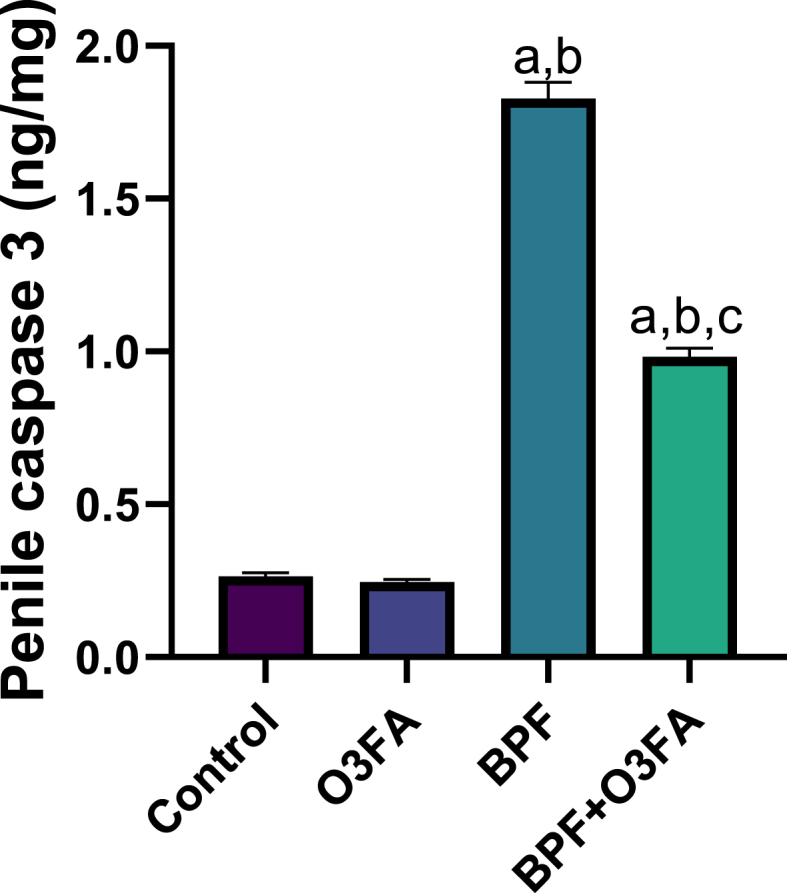
Effect of omega 3 fatty acids on penile caspase 3 in BPF-exposed rats. ^a^p <0 .05 versus control, ^b^p < 0.05 versus O3FA, ^c^p < 0.05 versus BPF using one-way analysis of variance (ANOVA) followed by Tukey's post hoc test for pairwise comparison. BPF: Bisphenol F, O3FA: omega-3 fatty acids.

Furthermore, BPF exposure significantly elevated penile caspase 3 in animals in the BPF group compared with their counterparts in the control groups (Fig. 9). The observed increase in this executioner caspase was blunted in animals in the BPF + O3FA group.

**Fig. 10:**
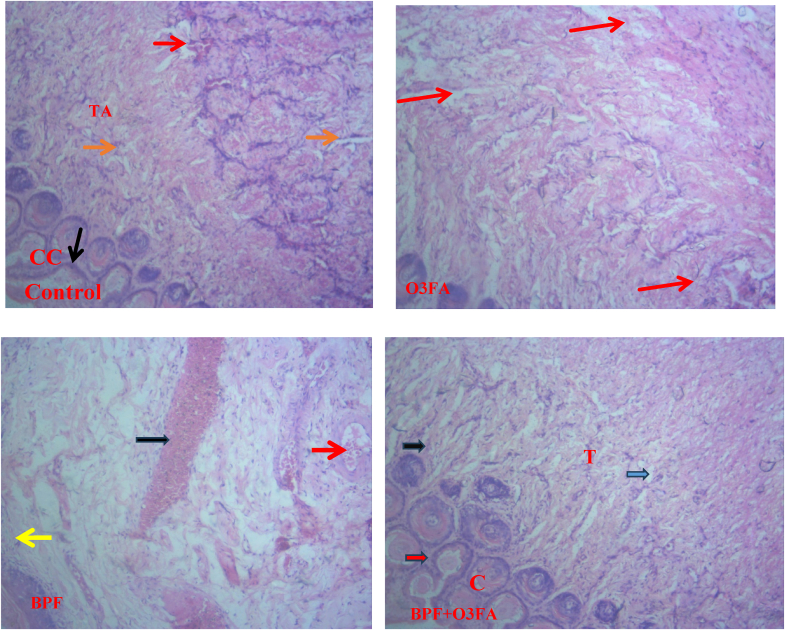
Histology of the penis (x100**). Control**: Phtomicrograph showed the normal histo-architecturer structure of the penis. Slide showed proper arrangement of the corpus cavernosal (CC), cavernosal sinuses lined by the endothelial cells (indicated with orange and black arrows), surrounding tunica albuginea (TA) and deep cavernosa artery (indicated with red arrow). O3FA: Photomicrograph showed the normal histo-architectural structure of the penis. The erectile tissues, corpus cavernosa (CC) and corpus spongosium (CS) are well preserved cavernosa sinuses lined by the endothelial cells (indicated with red arrows), surrounding tunica albuginea (TA). **BPF:** Photomicrograph showed atrophy in smooth muscle cells (SMC) within the corpora cavernosa. There is a decreased vasodilation of the cavernosa/deep artery (indicated with red arrow), shrinkage in the sinusoidal spaces (indicated with yellow arrow) and vascular congestion (indicated with black arrow). BPF + O3FA: Photomicrograph showed well structured histoarchitecture of the penis with normal sizes sinusoids, smooth muscle cells (indicated with black arrow), endothelial cells. The corpora cavernosa and its supporting tunica albuginea appeared normal. Reduced fat deposits (indicated with blue arrow) was also observed in the section. Corpora cavernosa (CC), tunica albuginea (TG). BPF: Bisphenol F, O3FA: Omega-3 fatty acids.

Finally, BPF disrupted normal penile cytoarchitecture evidenced by the presence of atrophied smooth muscle cells within the corpora cavernosa. There was also a decreased vasodilation of the cavernosal/deep artery, shrinkage in the sinusoidal spaces, and vascular congestion (Fig. 10). These observed alterations were ameliorated in the animals treated with BPF and O3FA.

## 4 Discussion

This study is the first study to explore the modulatory potential of O3FA on BPF-induced erectile dysfunction by maintaining redox balance and upregulating erectogenic enzymes. We found that BPF notably stunted erectogenic markers (dopamine, NO, cGMP, testosterone) and upsurge the anti-erectogenic enzymatic markers (serotonin, MAO, arginase, and PDE-5). Furthermore, BPF modulated penile XO/UA and Nrf2/Nf-κb signaling which led to an increase in penile MDA content and a decrease in non-enzymatic and enzymatic antioxidants (SOD, catalase, and GSH) content. This observed redox imbalance possibly triggered the observed inflammatory response (evidenced by an increase in penile IL-1β, Tnf-α, and MPO) and apoptotic response (increased penile caspase 3). Interestingly, the observed biochemical markers were supported by distortions in penile histoarchitecture. This reveals that BPF-induced erectile dysfunction via multiple pathways. Notably, O3FA ameliorated BPF-induced erectile dysfunction by upregulating the erectogenic markers and maintaining penile inflammatory and redox homeostasis.

Male sexual behavior requires sexual motivation and arousal which are two distinct phenomena [32]. Sexual arousal is maintained by the neural networks within the brainstem that regulate the behavioral and autonomic nervous system responses leading to penile erection while the sexual motivation drives maintains the subsequent stages of sexual behavior [33]. Although cavernosal electro-stimulation is an objective assessment of erectile function, it does not mimic the real-time penile erectile events in humans that are majorly stimulated by tactile, visual, and olfactory stimuli. Hence, the observed decrease in contact and non-contact (elicited by auditory, visual, and olfactory) reflexes and motivation to mate following BPF exposure are indicators of impaired male sexual behavior since the motivation to mate indicates sexual motivation while penile reflexes indicate sexual arousal which are the activities required for a fully expressed male sexual behavior [33]. These findings are in keeping with our previously reported observations that BPF exposure led to sexual and erectile dysfunction [7].

Although motivation to mate and penile reflexes are key indicators of male sexual behavior, penile erection is maintained by the activities of the NO/cGMP signaling [34]. This signaling is activated on sexual stimulation leading to the release of NO from the parasympathetic nerves into the smooth muscle cells present in the arteries of the corporal cavernosal. This released NO in turn activates the guanylate cyclase responsible for converting guanosine triphosphate to cGMP which is responsible for relaxing the carvernosal smooth muscle cells and increasing penile blood flow to bring about penile erection [24]. Hence, the observed decrease in NO/cGMP signaling activities following BPF exposure probably accounted for the earlier observed BPF-induced erectile dysfunction. Furthermore, BPF-induced NO/cGMP dysfunction is associated with increased PDE-5 enzymatic activities. PDE5 is a phosphodiesterase enzyme and it is responsible for rapid hydrolysis of cGMP thereby downregulating the activities of NO/cGMP signaling [35]. Also, the decline in NO/cGMP signaling following BPF exposure is associated with an increase in penile arginase. This increase in penile arginase will naturally compete with nitric oxide synthase for l-arginine [36,37], thereby decreasing NO production and in turn downregulating the NO/cGMP signaling. Therefore, the observed decline in cGMP levels in BPF-exposed rats may be attributed to BPF-driven increase in PDE-5 and arginase, and these could explain the observed impaired penile erectile function seen in BPF-exposed rats as cGMP is responsible for trabecular smooth muscle relaxation and increased penile blood flow leading to penile erection.

Furthermore, dopamine is a major monoamine neurotransmitter that plays a key role in male sexual and erectile function by mediating sexual motivation, arousal, and the consummatory aspects of sexual behavior [38]. Also, previous findings on laboratory animals revealed the classic dichotomy dopamine-serotonin relationship, with dopamine having a stimulatory and serotonin having an inhibitory effect on male sexual behavior [39,40]. Dopamine stimulates penile erection via dopamine-oxytocin-NO signaling which is responsible for activating NO [41]. Therefore, the observed decrease in dopamine and increased serotonin concentration following BPF treatment possibly accounts for, at least in part, BPF-induced erectile dysfunction. This observed decrease in dopamine could result from BPF-induced increase in penile MAO which is responsible for reducing the concentration of monoamines. In addition, control of normal sexual and erectile function is a matter of testosterone-dopamine crosstalk. Circulatory testosterone level within the normal range is important for dopamine synthesis and release [24,42], which is responsible for stimulating sexual behavior and penile erection. The findings from this study that BPF disrupts the monoamine neurotransmitter activities are in tandem with our previous findings [7] and that of Castro et al. [43].

Furthermore, redox balance plays a key role in endothelial function maintenance and penile homeostasis. A distortion in this redox balance leads to oxidative stress. Hence, oxidative stress impairs endothelial function, decreases the bioavailability of NO, and enhances vascular dysfunctions which are conditions that are associated with erectile dysfunction onset and progression [10]. In fact, the importance of endogenous antioxidants in maintaining penile homeostasis and oxidative stress has been established. Enzymatic and non-enzymatic antioxidants such as SOD, CAT, and GSH have been shown to be vital for the bioavailability of NO. Their effects are majorly achieved by shielding NO from being scavenged by superoxide radicals [44]. Furthermore, oxidative stress can reduce NO-mediated protein kinase G activation and VASP-TRPC4 interactions leading to the inability of NO to reduce calcium entry into the smooth cells muscle [45], thereby inhibiting penile erection. Additionally, oxidative stress can impair the dopaminergic system by oxidizing dopamine-derived catechols to produce reactive dopamine quinones which can further lead to oxidative stress [46]. Therefore, the observed increase in penile pro-oxidants and decrease in antioxidant activities following BPF exposure probably accounted for the observed NO/cGMP signaling dysfunction and monoamine neurotransmitter distortion. The observed redox imbalance in BPF-treated rats could account for the observed increase in inflammatory (Il-1β, TnF-α, and MPO) and apoptotic (caspase 3) markers since oxidative stress can lead to inflammation and then apoptosis [47,48]. This observed BPF-induced oxido-inflammatory response could be associated with Nrf2/Nf-κb and XO/UA signaling activities.

Nrf2 is a cytoprotective and a major transcription agent responsible for regulating numerous cell homeostasis by preventing oxidative stress [49]. It also acts as an anti-inflammatory agent by suppressing Nf- κb, a major regulator of inflammatory responses [[50], [51]]. Furthermore, the increase observed in XO following BPF exposure and the consequent increase in UA are markers of lipid peroxidation. While UA is a known antioxidant, it however becomes a pro-oxidant when produced in excess, thereby leading to ROS generation [51]. This excessive ROS can overwhelm the activities of Nrf2 and consequently lead to overexpression of Nf- κb leading to the inflammatory response. Hence, Nrf2/Nf-κb and XO/UA signaling played a key role in the observed penile redox imbalance following BPF exposure.

Furthermore, the novelty of the current study extends beyond demonstrating the involvement of penile redox imbalance in BPF-induced erectile dysfunction. Another key finding of this study is the therapeutic effect of O3FA in BPF and O3FA co-treated rats. The current study demonstrated that O3FA abrogated BPF-induced sexual and erectile dysfunction through inhibition of penile MAO, serotonin, arginase, and PDE-5 activities, and an increase in the level of NO, cGMP, dopamine, and testosterone. O3FA co-treatment with BPF also averted BPF-induced penile redox imbalance by inhibiting the activities of penile XO, UA, Nf-κb and upregulating Nrf2 level.

## 5 CONCLUSION

Findings from this study provide a new molecular insight into the protective molecular mechanisms of O3FA on BPF-induced sexual and erectile dysfunction. Our results revealed that O3FA co-treatment suppressed oxidative stress and inflammatory response by modulating Nrf2/Nf-κb and XO/UA signaling in BPF-treated animals. This was followed by the modulation of erectogenic mediators and consequently improving sexual and erectile function in BPF and O3FA-treated rats.

## CRediT authorship contribution statement

**Adeyemi Fatai Odetayo:** Writing – review & editing, Writing – original draft, Visualization, Validation, Supervision, Software, Resources, Project administration, Methodology, Investigation, Funding acquisition, Formal analysis, Data curation, Conceptualization. **Moses Agbomhere Hamed:** Writing – review & editing, Validation, Supervision, Software, Resources, Project administration, Methodology, Investigation, Funding acquisition, Formal analysis, Conceptualization. **Grace Edet Bassey:** Writing – review & editing, Visualization, Validation, Software, Resources, Project administration, Methodology, Investigation, Funding acquisition, Formal analysis. **Oluranti Olayinka Titiloye:** Writing – review & editing, Validation, Supervision, Resources, Project administration, Methodology, Investigation, Funding acquisition, Formal analysis. **Samson Daniel Maduabuchi:** Writing – review & editing, Software, Resources, Project administration, Methodology, Investigation, Funding acquisition. **Kazeem Bidemi Okesina:** Writing – review & editing, Supervision, Resources, Project administration, Methodology, Investigation, Funding acquisition. **Luqman Aribidesi Olayaki:** Writing – review & editing, Validation, Supervision, Software, Resources, Project administration, Methodology, Investigation, Funding acquisition, Formal analysis, Conceptualization.

## Funding

None.

## Declaration of competing interest

The authors declare that they have no known competing financial interests or personal relationships that could have appeared to influence the work reported in this paper.

## Data Availability

Data will be made available on request.

## Appendix A Supplementary data

Multimedia Component1 Multimedia Component1

## References

[1] Kumar M., Sarma D.K., Shubham S., Kumawat M., Verma V., Prakash A., Tiwari R. (2020). Environmental endocrine-disrupting chemical exposure: role in non-communicable diseases. Front. Public Health.

[2] Diamanti-Kandarakis E., Bourguignon J.P., Giudice L.C., Hauser R., Prins G.S., Soto A.M., Zoeller R.T., Gore A.C. (2009). Endocrine-disrupting chemicals: an endocrine society scientific statement. Endocr. Rev..

[3] Rybczyńska-Tkaczyk K., Skóra B., Szychowski K.A. (2023). Toxicity of bisphenol A (BPA) and its derivatives in divers biological models with the assessment of molecular mechanisms of toxicity. Environ. Sci. Pollut. Res. Int..

[4] Odetayo A.F., Adeyemi W.J., Olayaki L.A. (2023). In vivo exposure to bisphenol F induces oxidative testicular toxicity: role of Erβ and p53/Bcl-2 signaling pathway. Front. Reprod. Health.

[5] Odetayo A.F., Adeyemi W.J., Olayaki L.A. (2023). Omega-3 fatty acid ameliorates bisphenol F-induced testicular toxicity by modulating Nrf2/NFkB pathway and apoptotic signaling. Front. Endocrinol..

[6] Li B., Huo S., Du J., Zhang X., Zhang J., Song M., Li Y. (2025). Effect of bisphenol F on reproductive function in F1 generation Male mice and its potential mechanisms. Environ. Pollut..

[7] Odetayo A.F., Olayaki L.A. (2023). Omega 3 fatty acid improves sexual and erectile function in BPF-treated rats by upregulating NO/cGMP signaling and steroidogenic enzymes activities. Sci. Rep..

[8] Afzal S., Abdul Manap A.S., Attiq A., Albokhadaim I., Kandeel M., Alhojaily S.M. (2023). From imbalance to impairment: the central role of reactive oxygen species in oxidative stress-induced disorders and therapeutic exploration. Front. Pharmacol..

[9] Manchope M.F., Calixto-Campos C., Coelho-Silva L., Zarpelon A.C., Pinho-Ribeiro F.A., Georgetti S.R., Baracat M.M., Casagrande R., Verri W.A. (2016). Naringenin inhibits superoxide anion-induced inflammatory pain: role of oxidative stress, cytokines, Nrf-2 and the NO-cGMP-PKG-KATP channel signaling pathway. PLoS One.

[10] Kaltsas A., Zikopoulos A., Dimitriadis F., Sheshi D., Politis M., Moustakli E., Symeonidis E.N., Chrisofos M., Sofikitis N., Zachariou A. (2024). Oxidative stress and erectile dysfunction: pathophysiology, impacts, and potential treatments. Curr. Issues Mol. Biol..

[11] Konstantinopoulos A., Giannitsas K., Raptis S., Perimenis P. (2007). Endothelial dysfunction, erectile dysfunction and phosphodiesterase 5 inhibitors. An update of the current data and future perspectives. Drug Target Insights.

[12] Yarak N., El Khoury J., Coloby P., Bart S., Abdessater M. (2024). Idiopathic recurrent ischemic priapism: a review of current literature and an algorithmic approach to evaluation and management. Basic Clinical Andrology.

[13] Swanson D., Block R., Mousa S.A. (2012). Omega-3 fatty acids EPA and DHA: health benefits throughout life. Adv. Nutr..

[14] Sherratt S.C.R., Libby P., Budoff M.J., Bhatt D.L., Mason R.P. (2023). Role of Omega-3 fatty acids in cardiovascular disease: the debate continues. Curr. Atheroscler. Rep..

[15] Odetayo A.F., Abdulrahim H.A., Fabiyi O.T., Adewole T.A., Ajiboye B.E., Omeiza A.N., Olayaki L.A. (2024). Cell Biochemistry and Biophysics.

[16] Fernández-Lázaro D., Arribalzaga S., Gutiérrez-Abejón E., Azarbayjani M.A., Mielgo-Ayuso J., Roche E. (2024). Omega-3 fatty acid supplementation on post-exercise inflammation, muscle damage, oxidative response, and sports performance in physically healthy Adults-A systematic review of randomized controlled trials. Nutrients.

[17] Fatai O.A., Aribidesi O.L. (2022). Effect of bisphenol F on sexual performance and quality of offspring in Male wistar rats. Ecotoxicol. Environ. Saf..

[18] Odetayo A.F., Olayaki L.A. (2022). Bisphenol F induced reproductive toxicity by disrupting steroidogenic enzymes activities and upregulating xanthine oxidase/uric acid signaling. Fertil. Steril..

[19] Higashihara N., Shiraishi K., Miyata K., Oshima Y., Minobe Y., Yamasaki K. (2007). Subacute oral toxicity study of bisphenol F based on the draft protocol for the "Enhanced OECD Test Guideline no. 407". Arch. Toxicol..

[20] Huitao L., Jingjing L., Lei S., Yang Z., Fuhong T., Mengna S., Qiyao L., Ren-shan G. (2022). Bisphenol F blocks leydig cell maturation and steroidogenesis in pubertal Male rats through suppressing androgen receptor signaling and activating G-protein coupled estrogen receptor 1 (GPER1) signaling. Food Chem. Toxicol..

[21] Odetayo A.F., Olayaki L.A. (2024). Omega 3 fatty acids preserve testicular function by ameliorating BPF-induced dysthyroidism: role of p53/Bcl-2 signaling and proton pump activities. JBRA Ass.Reproduct..

[22] Jimmy E.O., Bassey G.E., Ekwere A., Umoh U.I., Odetayo A.F., Malachy U.N. (2025). Finger roots (Uvaria chemea) and African greenheart (Cylicodiscus gabunensis): alternative potent therapy to sildenafil in erectile dysfunction. Phytomed. Plus.

[23] Akhigbe R.E., Hamed M.A., Odetayo A.F. (2021). HAART and anti-koch's impair sexual competence, sperm quality and offspring quality when used singly and in combination in male wistar rats. Andrologia.

[24] Akhigbe R.E., Hamed M.A., Odetayo A.F., Akhigbe T.M., Oyedokun P.A. (2023). Zinc improves sexual and erectile function in HAART-treated rats via the upregulation of erectogenic enzymes and maintenance of redox balance. Aging Male : Off. J. Int. Soc. Study Aging Male.

[25] Kettler R., Da Prada M., Burkard W.P. (1990). Comparison of monoamine oxidase-A inhibition by moclobemide in vitro and ex vivo in rats. Acta psychiatrica Scandinavica. Supplementum.

[26] Kelly S.J., Butler L.G. (1977). Enzymic hydrolysis of phosphonate esters. Reaction mechanism of intestinal 5'-nucleotide phosphodiesterase. Biochemistry.

[27] Zhang C., Hein T.W., Wang W., Chang C.I., Kuo L. (2001). Constitutive expression of arginase in microvascular endothelial cells counteracts nitric oxide-mediated vasodilatory function. FASEB J. : Off. Pub. Feder. Am. Soc. Exp. Bio..

[28] Beutler E., Duron O., Kelly B.M. (1963). Improved method for the determination of blood glutathione. J. Lab. Clin. Med..

[29] Misra H.P., Fridovich I. (1972). The role of superoxide anion in the autoxidation of epinephrine and a simple assay for superoxide dismutase. J. Biol. Chem..

[30] Euler H.V., Josephson K. (1927). Über katalase. I. Justus Liebigs Ann. Chem..

[31] Desser R.K., Himmelhoch S.R., Evans W.H., Januska M., Mage M., Shelton E. (1972). Guinea pig heterophil and eosinophil peroxidase. Arch. Biochem. Biophys..

[32] Sachs B.D. (2000). Contextual approaches to the physiology and classification of erectile function, erectile dysfunction, and sexual arousal. Neurosci. Biobehav. Rev..

[33] Bialy M., Bogacki-Rychlik W., Przybylski J., Zera T. (2019). The sexual motivation of Male rats as a tool in animal models of human health disorders. Front. Behav. Neurosci..

[34] Boolell M., Allen M.J., Ballard S.A., Gepi-Attee S., Muirhead G.J., Naylor A.M., Osterloh I.H., Gingell C. (1996). Sildenafil: an orally active type 5 cyclic GMP-specific phosphodiesterase inhibitor for the treatment of penile erectile dysfunction. Int. J. Impot. Res..

[35] Andersson K.E. (2018). PDE5 inhibitors - pharmacology and clinical applications 20 years after sildenafil discovery. Br. J. Pharmacol..

[36] Parvardeh S., Sabetkasaei M., Moghimi M., Masoudi A., Ghafghazi S., Mahboobifard F. (2018). Role of L-arginine/NO/cGMP/K_ATP_ channel signaling pathway in the central and peripheral antinociceptive effect of thymoquinone in rats. Iran. J. Med. Sci..

[37] Benza R.L., Grünig E., Sandner P., Stasch J.P., Simonneau G. (2024). The nitric oxide-soluble guanylate cyclase-cGMP pathway in pulmonary hypertension: from PDE5 to soluble guanylate cyclase. Eur. Respir. Rev. : Off. J. Europ. Resp. Soc..

[38] Melis M.R., Sanna F., Argiolas A. (2022). Dopamine, erectile function and Male sexual behavior from the past to the present: a review. Brain Sci..

[39] Hull E.M., Muschamp J.W., Sato S. (2004). Dopamine and serotonin: influences on male sexual behavior. Physiol. Behav..

[40] Graf H., Malejko K., Metzger C.D., Walter M., Grön G., Abler B. (2019). Serotonergic, dopaminergic, and noradrenergic modulation of erotic stimulus processing in the Male human brain. J. Clin. Med..

[41] Koon C.S., Sidi H., Kumar J., Xi O.W., Das S., Hatta M.H., Alfonso C. (2018). The phosphodiasterase 5-Inhibitors (PDE-5i) for Erectile Dysfunction (ED): a therapeutic challenge for psychiatrists. Curr. Drug Targets.

[42] Purves-Tyson T.D., Handelsman D.J., Double K.L., Owens S.J., Bustamante S., Weickert C.S. (2012). Testosterone regulation of sex steroid-related mRNAs and dopamine-related mRNAs in adolescent Male rat substantia nigra. BMC Neurosci..

[43] Castro B., Sánchez P., Torres J.M., Ortega E. (2015). Bisphenol A, bisphenol F and bisphenol S affect differently 5α-reductase expression and dopamine-serotonin systems in the prefrontal cortex of juvenile female rats. Environ. Res..

[44] Roychoudhury S., Chakraborty S., Choudhury A.P., Das A., Jha N.K., Slama P., Nath M., Massanyi P., Ruokolainen J., Kesari K.K. (2021). Environmental factors-induced oxidative stress: Hormonal and molecular pathway disruptions in hypogonadism and erectile dysfunction. Antioxidants.

[45] Banday A.A., Lokhandwala M.F. (2019). Oxidative stress impairs cGMP-dependent protein kinase activation and vasodilator-stimulated phosphoprotein serine-phosphorylation. Clin. Exp. Hypertens..

[46] Girotto S., Sturlese M., Bellanda M., Tessari I., Cappellini R., Bisaglia M., Bubacco L., Mammi S. (2012). Dopamine-derived quinones affect the structure of the redox sensor DJ-1 through modifications at Cys-106 and Cys-53. J. Biol. Chem..

[47] Dmytriv T.R., Duve K.V., Storey K.B., Lushchak V.I. (2024). Vicious cycle of oxidative stress and neuroinflammation in pathophysiology of chronic vascular encephalopathy. Front. Physiol..

[48] Abdulrahim H.A., Odetayo A.F., Owootori E.A., Bulus J.D., Jimoh F.B., Gabriel E.O., Odiete I.F., Olayaki L.A. (2024). Naunyn-schmiedeberg's Archives of Pharmacology.

[49] Ajibare A.J., Odetayo A.F., Akintoye O.O., Oladotun A.J., Hamed M.A. (2024). Zinc abates sodium benzoate-induced testicular dysfunction via upregulation of Nrf2/HO-1/Nf-κB signaling and androgen receptor gene. J. Trace Elem. Med. Biol.: Organ Soc. Minerals Trace Elements (GMS).

[50] Hamed M.A., Akhigbe R.E., Aremu A.O., Odetayo A.F. (2022). Zinc normalizes hepatic lipid handling via modulation of ADA/XO/UA pathway and caspase 3 signaling in highly active antiretroviral therapy-treated wistar rats. Chem. Biol. Interact..

[51] Okesina K.B., Odetayo A.F., Adeyemi W.J., Ajibare A.J., Okesina A.A., Olayaki L.A. (2024). Naringin from sweet orange peel improves testicular function in high fat diet-induced diabetic rats by modulating xanthine oxidase/uric acid signaling and maintaining redox balance. Lab. Animal Res..

