# Supplementary material for: Prophylactic role of omega 3 fatty acids in bisphenol F-induced sexual and erectile dysfunction is associated with penile redox homeostasis
Source: Biochem Biophys Rep. 2025 Jun 23;43:102103. doi: 10.1016/j.bbrep.2025.102103 (PMC12242445; doi:10.1016/j.bbrep.2025.102103)
Supplement: Multimedia Component1 [file mmc1.docx]

**Prophylactic role of omega 3 fatty acids in bisphenol F-induced sexual and erectile dysfunction is associated with penile redox homeostasis**

Adeyemi Fatai Odetayo^1,2^*, Moses Agbomhere Hamed^3,4*^, Grace Edet Bassey^5^, Oluranti Olayinka Titiloye^6^, Samson Daniel Maduabuchi^7^, Kazeem Bidemi Okesina^8^Luqman Aribidesi Olayaki^9^

^1^Department of Physiology, Faculty of Basic Medical Sciences, Federal University of Health Sciences, Ila-Orangun, Nigeria

^2^Endocrinology, Reproductive, and Metabolism Unit, Physiology Department, Federal University of Health Sciences, Ila-Orangun, Nigeria

^3^Department of Medical Laboratory Science, Afe Babalola University, Ado-Ekiti, Nigeria.

^4^The Brainwill Laboratories and Biomedical Services, Osogbo, Nigeria

^5^Physiology Department, Faculty of Basic Medical Sciences, University of Uyo, Uyo, Nigeria

^6^Department of Anatomy and Cell Biology, Obafemi Awolowo University, Ife, Nigeria

^7^Department of Medical Laboratory Sciences, Leeds City University, Ibadan, Nigeria

^8^Department of Physiology, University of Rwanda, Kigali, Rwanda

^9^Department of Physiology, University of Ilorin, Ilorin, Nigeria

*Corresponding Author:

Kazeem Bidemi Okesina

Email address: [o.bidemi@ur.ac.rw](mailto:o.bidemi@ur.ac.rw) or flexability777@gmail.com

Adeyemi Fatai Odetayo

[Adeyemi.odetayo@fuhsi.edu.ng](mailto:Adeyemi.odetayo@fuhsi.edu.ng) or adeyemiodetayo@gmail.com

2347032766035

Running Title: Omega-3 Fatty Acids improved erectile function in bisphenol F-induced penile dysfunction

Supplementary Table 1: List of previous studies on BPF

| Authors | DOI | Major findings | Novelty of our findings |
| --- | --- | --- | --- |
| Ullah et al., 2019 | <https://doi.org/10.1177/0960327119862335> | BPA and its analogs BPB, BPF, and BPS at different concentrations disrupted testicular functions |  |
| Lee et al., 2022 | [doi.org/10.1016/j.yrtph.2022.105286](https://doi.org/10.1016/j.yrtph.2022.105286) | BPF impaired female reproduction functions and pregnancy outcomes |  |
| Fatai and Luqman, 2022 | [10.1016/j.ecoenv.2022.114079](https://doi.org/10.1016/j.ecoenv.2022.114079) | BPF impaired male sexual performance and pregnancy outcomes |  |
| Odetayo et al., 2023 | 10.3389/fendo.2023.1256154 | Omega-3 fatty acids prevented BPF-induced testicular toxicity |  |
| Odetayo and Olayaki, 2023 | [10.1038/s41598-023-45344-4](https://doi.org/10.1038/s41598-023-45344-4) | Omega-3 fatty acids prevented BPF-induced sexual dysfunction |  |
| Odetayo and Olayaki, 2024 | [10.5935/1518-0557.20240033](https://doi.org/10.5935/1518-0557.20240033) | BPF-induced testicular dysfunction was reported to be associated with BPF-induced dysthyroidism |  |
| Higley et al., 2024 | [doi.org/10.1177/07482337241287](https://doi.org/10.1177/07482337241287967) | BPs adversely affect male fertility to varying degree |  |
| Wu et al., 2025 | [10.1016/j.taap.2025.117245](https://doi.org/10.1016/j.taap.2025.117245) | BPF exposure impairs sperm quality and offspring development in male zebrafish |  |
| Li et al., 2025 | [doi.org/10.1016/j.envpol.2024.125546](https://doi.org/10.1016/j.envpol.2024.125546) | Gestational exposure to BPF can lead to reproductive dysfunction in F1 generation male mice |  |
| Shi et al., 2024 | [doi.org/10.1016/j.envpol.2024.124531](https://doi.org/10.1016/j.envpol.2024.124531) | FTO mediates bisphenol F-induced blood-testis barrier impairment through regulating ferroptosis via YTHDF1/TfRc and YTHDF2/SLC7A11 signal axis |  |
